# Optical simulations of P3HT/Si nanowire array hybrid solar cells

**DOI:** 10.1186/1556-276X-9-238

**Published:** 2014-05-14

**Authors:** Wenbo Wang, Xinhua Li, Long Wen, Yufeng Zhao, Huahua Duan, Bukang Zhou, Tongfei Shi, Xuesong Zeng, Ning Li, Yuqi Wang

**Affiliations:** 1 Key Laboratory of Material Physics, Institute of Solid State Physics, Chinese Academy of Sciences, Hefei 230031, China; 2 Key Laboratory of Nanodevices and Applications, Suzhou Institute of Nano-Tech and Nano-Bionics, Chinese Academy of Sciences, Suzhou 215123, China

**Keywords:** P3HT, Si nanowire array, Hybrid solar cells, Finite-difference time-domain (FDTD) method

## Abstract

An optical simulation of poly(3-hexylthiophene) (P3HT)/Si nanowire array (NWA) hybrid solar cells was investigated to evaluate the optical design requirements of the system by using finite-difference time-domain (FDTD) method. Steady improvement of light absorption was obtained with increased P3HT coating shell thickness from 0 to 80 nm on Si NWA. Further increasing the thickness caused dramatic decrease of the light absorption. Combined with the analysis of ultimate photocurrents, an optimum geometric structure with a coating P3HT thickness of 80 nm was proposed. At this structure, the hybrid solar cells show the most efficient light absorption. The optimization of the geometric structure and further understanding of the optical characteristics may contribute to the development for the practical experiment of the promising hybrid solar cells.

## Background

Solar cells based on polymer materials provide a promising route toward cost-effective, large-area, and flexible organic photovoltaic (OPV) solar cells [1-3]. Among all the photoactive polymer materials, poly(3-hexylthiophene) (P3HT) is one of the most widely used photoactive materials in fabricating organic solar cells. Considerable efforts have been focused on enhancing the energy conversion efficiency, where the best devices consist of a mixture of polythiophene derivatives (electron donor) and C60 fullerenes (electron acceptor), called a bulk heterojunction (BHJ) type, where the maximum power conversion efficiency (PCE) has reached approximately 7.5% to date [4]. Another promising way for facilitating carrier collection is to fabricate nanostructure-based hybrid solar cells that use ordered semiconductor nanowire array (NWA) surrounded by photoactive organics. Benefitted from the ease of fabrication and cost-effectiveness, Si NWA is utilized to form P3HT/Si NWA hybrid solar cells. Over standard hybrid solar cells, it is expected that the Si NWA-based solar cells have the following advantages: On the electrical side, due to high carrier mobility and small dimensions, the Si NWA offers straight pathways for the carriers to escape the device as quickly as possible [5]. On the optical side, the light absorption is extend to infrared below the bandgap of silicon, thereby more photons in the solar radiation can be harvested. Meanwhile, due to their sub-wavelength dimensions, the strong light trapping effects arising from light scattering, light guiding, and inherent antireflection properties make NWA constructed hybrid solar cells absorb more photons with less material consumption as compared with conventional planar structure [6-10].

Because of these advantages, researches focusing on hybrid solar cells of P3HT/Si NWA have been done by many groups [11,12]. In the past few years, the reported devices' performances have been improved, but the published PCE of P3HT/Si NWA solar cells are still low. From the published reports of other inorganic semiconductor solar cells based on NWA, the property, especially optical absorptivity, of the photovoltaic device depends critically on the geometry of the sub-wavelength NWA structure [13-15]. The absence of properly optimized structure may be the main reason for the low PCE of the proposed hybrid solar cells. Thus, before practical fabrication of P3HT/Si NWA hybrid solar cells, the geometry of P3HT/Si NWA must be optimized. In view of this, in this paper, we do an optical simulation about P3HT/Si NWA hybrid solar cells to explore the optical characteristics of the system, so as to give an optical guidance for the practical fabrication of P3HT/Si NWA hybrid solar cells.

## Methods

In this paper, an optical simulation about P3HT/Si NWA hybrid solar cells was investigated to explore the optical characteristics of the system. First, the influence of the thickness of P3HT on the optical absorption of solar cells has been thoroughly analyzed by using finite-difference time-domain (FDTD) method [16]. Second, to further understand the optical absorption of the system, the optical generation rates in the *x*-*z* cross section of hybrid P3HT/Si NWA under optimized coated and uncoated Si NWA were obtained. Finally, to find an optimized geometry, the ultimate photocurrents were calculated to maximize the light absorption capability of the hybrid solar cells in the solar spectrum.

**Figure 1:**
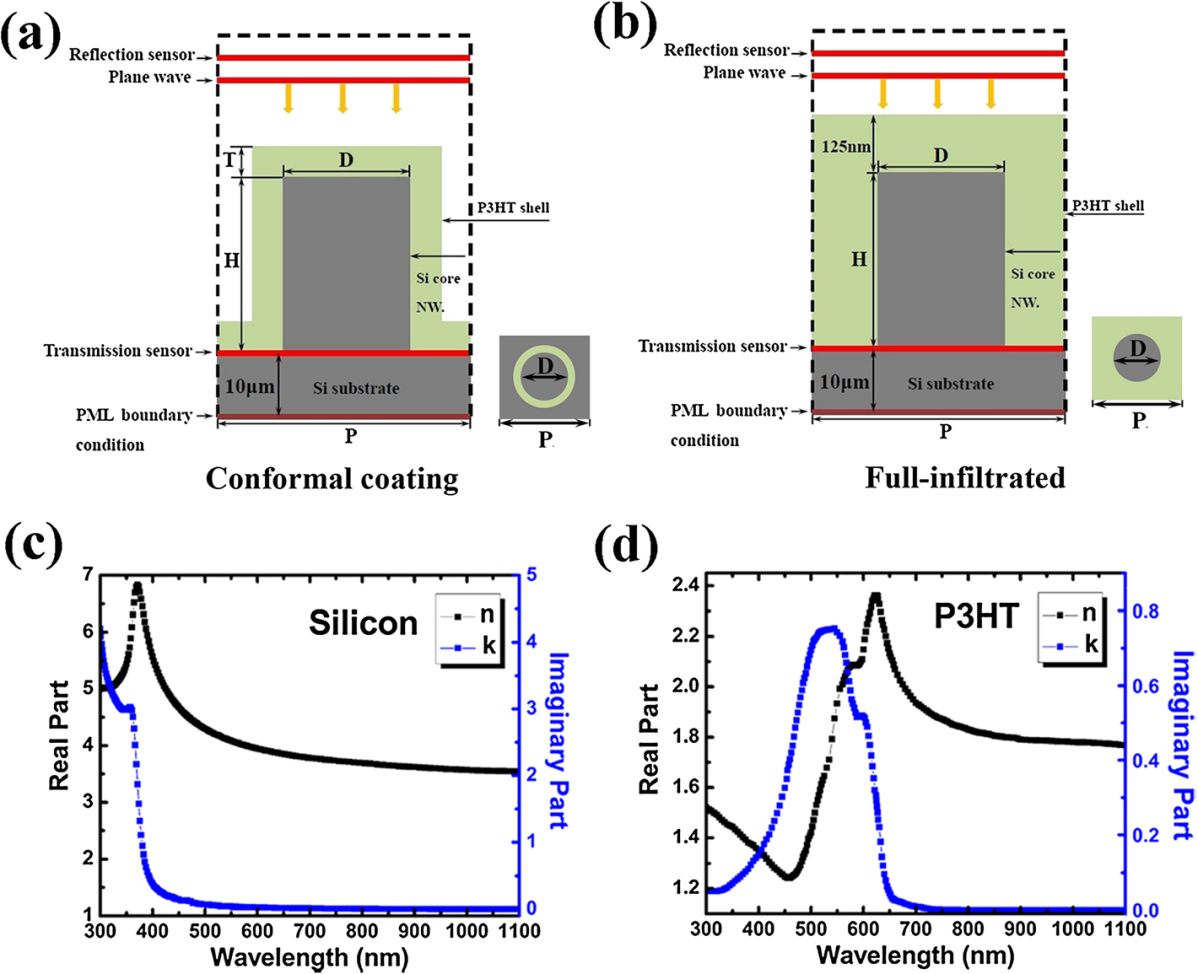
**Unit of P3HT/Si NWA hybrid solar cells and refractive indexes of silicon and P3HT. (a)** Simulated unit of P3HT/Si NWA hybrid solar cells modeled in this study: conformal coating. **(b)** Simulated unit of P3HT/Si NWA hybrid solar cells modeled in this study: full-infiltrated. **(c)** Refractive index of silicon. **(d)** Refractive index of P3HT.

Figure 1a shows the schematics of the simulated unit of the proposed hybrid solar cells, which comprised vertically aligned Si NWA coated with conformal thin layer of P3HT on supporting Si substrate. The simulated region of FDTD is represented by a dashed frame, in which perfect match layer (PML) boundary conditions as well as source are signed. Meanwhile, as shown in Figure 1b, a more realistic condition, under which the Si NWA structure is fully infiltrated with P3HT, is also considered. The refractive indexes of silicon and P3HT used in this simulation are shown in Figure 1c,d. The parameters of this structure are the period of the square lattice *P*, Si core NWs diameter *D*, NW's height *H*, and organic shell thickness *T*. By placing the periodic boundary conditions, the simulations were carried out in a unit cell to model the periodic square-array wire structure with substrate. In our simulation, the optimized geometry of silicon NWs on flat Si substrate was fixed as *P* = 500 nm, *D* (SI) = 250 nm, and *H* = 5 μm [14]. It has been confirmed that the Si NWA with this structure as mentioned has the most efficient light absorption. In order to simplify the calculation, the Si thin film is assumed infinitely thick with no transmission loss by using a PML adjacent underneath the Si film. Note that the transmission sensor was set at the bottom of Si NWA. Hence, the optical characteristics we discussed in the following sections are related to NWA (or P3HT/Si NWA). The absorption in the bottom Si substrate is not included. Meanwhile, the optical generation rates and ultimate photocurrents were also achieved to give an optical optimization and analysis of the proposed hybrid P3HT/Si NWA structure.

## Results and discussion

**Figure 2:**
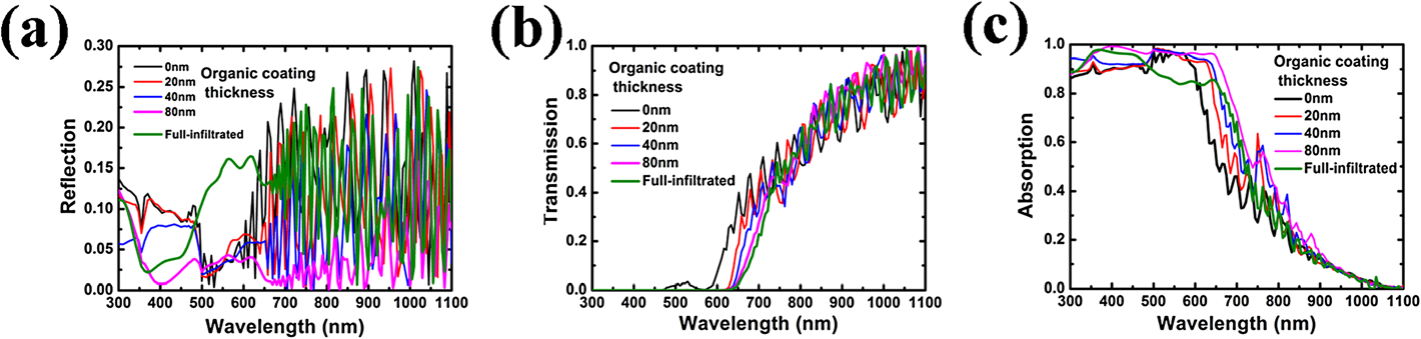
**Optical characteristics of the hybrid solar cells with various P3HT coating thicknesses. (a)** Reflection. **(b)** Transmission. **(c)** Absorption.

Figure 2a,b,c show the optical properties of P3HT/Si NWA hybrid system with various coating thicknesses of P3HT. As shown in Figure 2c, in the shorter wavelength region (<650 nm), one can find that the absorption of the P3HT/Si NWA system increases strongly as the thickness of the organic shell is increased. The absorptance of the NW/organic array reaches a maximum at the coating thickness of 80 nm. Further increasing the shell thickness will cause decrease of absorption, which is attributed to reflectance enhancement (Figure 2a) at this wavelength region. Due to the increase of photoactive material, the addition of organic coating can further decrease the transmission of P3HT/Si NWA structure in this wavelength region (Figure 2b). Meanwhile, as the thickness of the organic shell is increased to 80 nm, the reflection of P3HT/Si NWA system is suppressed, which stems mainly from the improved optical impedance matching between air and Si due to the presence of the additional low-index coating layer. Note that in the wavelength region from 500 to 580 nm, the absorption curve of P3HT/Si NWA (*T* = 40 and 80 nm) overlaps with that of bare Si NWA. This is due to the fact that the bare Si NWA exhibits the absorptance close to 1 in this wavelength region. Thus, although the absorptivity is increased as the P3HTs are coated on the surface of NWA, the absorption curves do not exhibit obvious enhancement. When the incident wavelength is above 650 nm, P3HT becomes transparent and only Si absorbs incident light. At this region, despite the size of photoactive Si NW is fixed, a certain amount of absorption enhancement can still be observed as the thickness of organic coating is increased. For example, at the wavelength of 700 nm, we note that the absorption at *T* = 80 nm has a factor of 1.81 higher than the case of the uncoated NWs. This can be understood by electrostatic approximation. The absorption in Si NW is proportional to the factor of |*E*_core_ / *E*_inc_|^2^, where *E*_core_ and *E*_inc_ are the electric field intensity in the core and incident light of Si NW, respectively [17]. In the absence of the organic coating, |*E*_core_ / *E*_inc_|^2^ = |2*ϵ*_ext_ / (*ϵ*_ext_ + *ϵ*_core_)|^2^ = 0.0169, where *ϵ*_ext_ = 1 is the dielectric function of the vacuum exterior to the NW, and *ϵ*_core_ ≈ 14.34 + 0.0985i is the dielectric function (for *λ* = 700 nm) of the Si NW. When an organic coating is added, |*E*_core_ / *E*_inc_|^2^ = |2*ϵ*_ext_ / (*ϵ*_ext_ + *ϵ*_coat_)|^2^|2*ϵ*_coat_ / (*ϵ*_coat_ + *ϵ*_core_)|^2^ = 0.030, where *ϵ*_coat_ = 3.75 is the dielectric function (for *λ* = 700 nm) of P3HT. About 1.78 times enhancement can be obtained at organic coating *T* = 80 nm than that of uncoated NWs, which is close to the absorptance enhancement at this wavelength (as shown in Figure 2c). Obviously, above the cutoff of P3HT, the organic coating can serve as a non-absorbing dielectric shell, which drastically increased the absorption in vertical semiconductor NWs. Moreover, at the wavelength larger than 650 nm, the extinction coefficient of silicon is small and interference effects exist, resulting in the oscillation of reflectance and transmittance [6].

**Figure 3:**
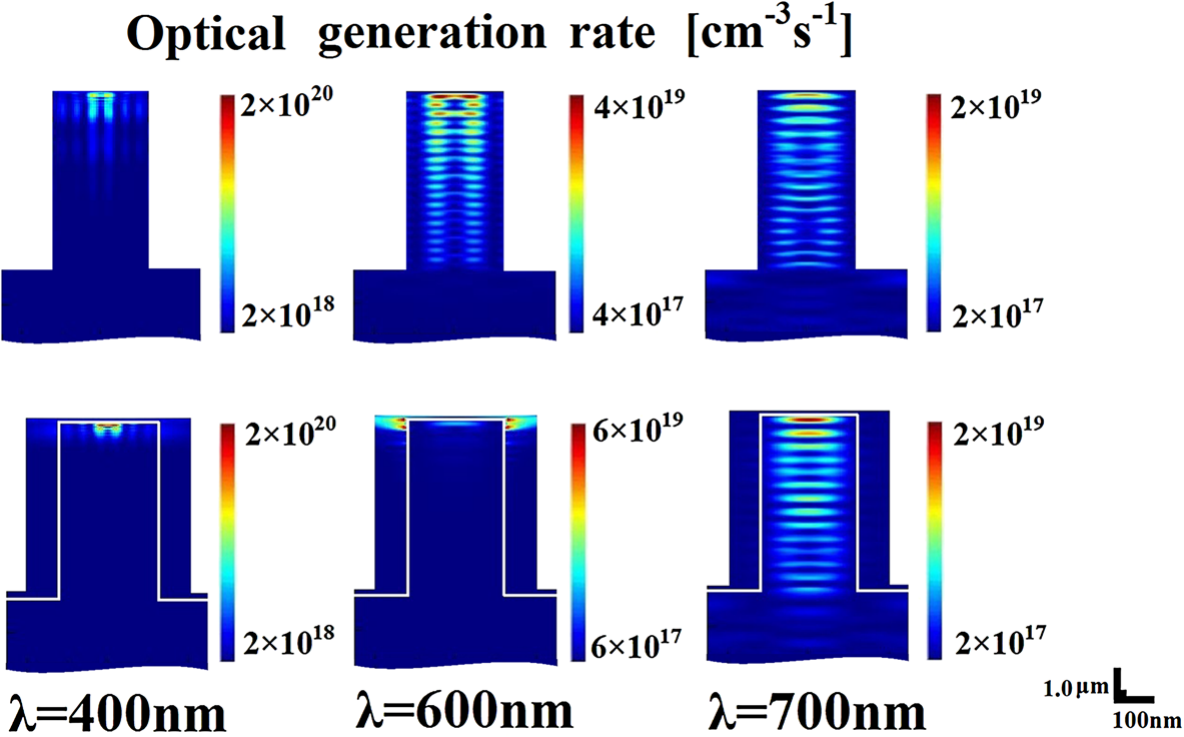
**Optical generation rates.** The wavelengths are 400, 600, and 700 nm for uncoated Si nanowire (above) and conformal coating hybrid structure (below).

In order to understand the propagation of light in the hybrid solar cells, we simulated the electrical field intensity and calculated the optical generation rates within the arrays from $G_{\mathrm{opt}}=\frac{\mathrm{real}\left(\nabla •S\right)}{2\hbar \omega }=\frac{\epsilon \prime \prime {\left|E\right|}^{2}}{2\hbar },$ where *ϵ*″ is the imaginary part of the complex permittivity and *E* is the electric field [18]. We give the optical generation rates for conformal coating hybrid structure with 80-nm P3HT at three typical wavelengths of 400, 600, and 700 nm. The optical generation rates of the uncoated Si NWs are used as comparison. As plotted in Figure 3, the optical generation rates at incident wavelengths of 400 and 600 nm are concentrated near the top and sides of the NW for conformal coating structure. For the uncoated Si NWs, different absorption patterns were obtained at wavelengths of 400 and 600 nm. For 400 nm, light absorption occurs mainly at the top part of the NW. At 600 nm, one can find that the optical generation rate exhibits more homogeneous spreading over the uncoated Si NWs and shows considerable oscillation absorption. At 700 nm, the optical generation rates are concentrated to several lobes that form along the Si NW for both structures, indicating strong guided modes confined inside the NWs. This phenomenon is similar to the absorption in Si NWs as reported by Lin and Povinelli [15]. Moreover, a small fraction of the incident wave is transmitted to the substrate for both structures at this wavelength. Comparatively, at the incident wavelength of 700 nm, a more intensive optical generation rate can be observed in Si NW with 80-nm organic coating than the case of uncoated Si NW, indicating a significant absorption enhancement of the non-absorbing dielectric shell.

From the above discussion, it is clear that the light absorption of the hybrid structure is quite sensitive to structural parameters. By proper choice of organic coating thickness, we find that the absorption of NWA is significantly enhanced. To further determine the optimized geometric configuration, the ultimate photocurrents were calculated for various thicknesses. We denoted the ultimate photocurrent by assuming perfect carrier extraction [19]: *J*_ph_ = (*e* / *hc*) *∫ λA*(*λ*)*I*(*λ*)*dλ*, where *e* is the elementary charge, *h* is Plank's constant, *c* is the light speed, *I*(*λ*) is the AM1.5G spectrum, and *A*(*λ*) is the absorption of the solar cells.

**Figure 4:**
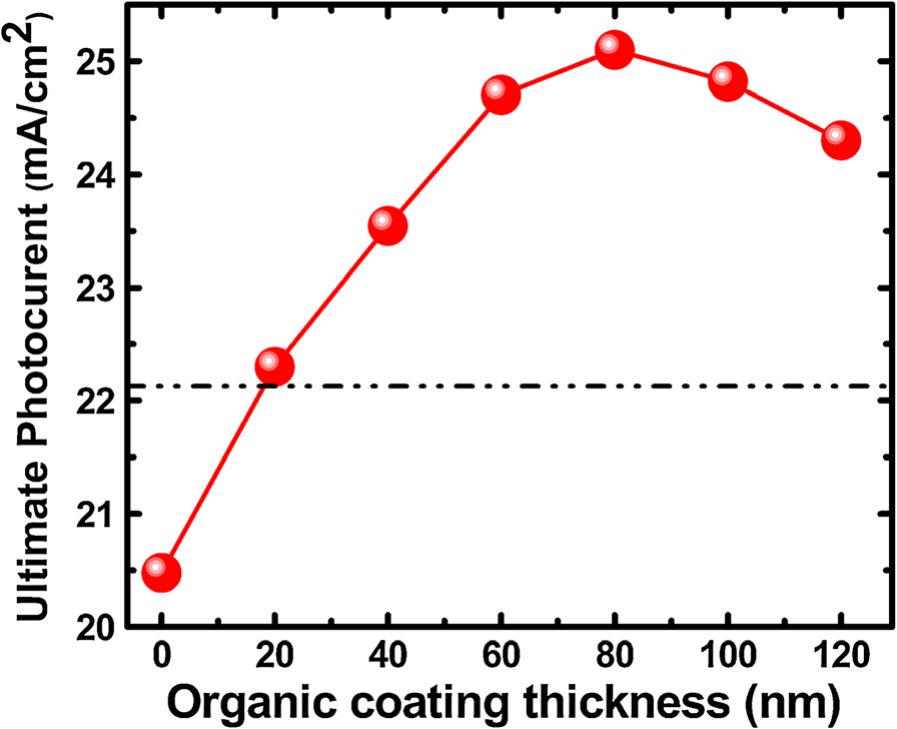
**Ultimate photocurrent as a function of organic coating thickness.** Dashed line indicates the value of full-infiltrated situation.

The ultimate photocurrent as a function of the coating thickness of P3HT is shown in Figure 4. The ultimate photocurrent is increase gradually with increasing organic coating thickness from 0 to 80 nm. The numerical value reaches a maximum of approximately 25 mA/cm^2^ at the coating shell thickness of 80 nm, which is 22% higher than that of the uncoated Si NWA. Further increasing the thickness of P3HT to 100 nm, 120 nm, and full infiltration causes a dramatic decrease of the ultimate photocurrent. The value signed with a dashed line in Figure 4 indicates the situation of full infiltration and gets an ultimate photocurrent of 22.2 mA/cm^2^. One can see that the ultimate photocurrent of full-infiltrated condition is about 3 mA/cm^2^ lower than that of the conformal coating condition of 80 nm. This shows the superiority performance of core-shell structure as compared with full-infiltrated condition. Obviously, great improved light absorption could be obtained, with appropriate coating organic thickness on the inorganic Si NWs.

From the discussion above, in the fabrication of P3HT/Si NWA hybrid solar cells, a conformal organic coating with thickness of 80 nm is proposed to be spun onto the surface of Si NWA to obtain the maximum optical absorption. But, this thickness is much larger than the exciton diffusion length (approximately 10 nm) in P3HT [20]. Recently, Paulus et al. have presented their experimental and theoretical results on nano-heterojunction organic solar cells, in which the maximum photocurrent occurs at 60 to 65 nm of a P3HT photoactive layer due to bulk exciton sink in P3HT [21,22]. Considering the P3HT/Si NWA hybrid structure has the same exciton dissociation mechanism as that proposed by Paulus et al., the thickness of the conformal P3HT thickness can be increased above the exciton diffusion length in the design of P3HT/Si NWA hybrid cells. Meanwhile, from Figure 4, good light absorption could still be maintained for a hybrid structure with a P3HT coating thickness slightly less than 80 nm. So, for practical fabrication of P3HT/Si NWA hybrid solar cells, the conformal coating with thickness of dozens of nanometers is propitious for the balance of the photon absorption, charge separation, and charge transport in the proposed P3HT/Si NWA hybrid solar cells.

## Conclusion

In conclusion, an optical simulation was investigated to evaluate the optical design requirements for improving the efficiency of P3HT/Si NWA solar cells. It is found that as a photoactive material, the introduction of organic coating on Si NWA can further increase the absorptance of P3HT/Si NWA hybrid structure, leading to a better light absorption for wavelengths both below and above the absorption cutoff wavelength of P3HT. At optimized size, the proposed hybrid solar cells exhibit promising photo absorption efficiency. Moreover, we give a direct theoretical proof about the superior performance of the core-shell condition with conformal coating of P3HT as compared with full-infiltrated condition. These findings will play a significant role in realizing the most effective hybrid solar cells formed by organic and semiconductor NWAs in practical experiment. Combined with easy and superior fabrication of such hybrid solar cells, a breakthrough in cell efficiency of the proposed device may be achieved. Obviously, the combination of low-cost Si NWA and solution-processed photoactive organic coating makes this P3HT/Si NWA hybrid solar cell worthy of further investigation.

## Abbreviations

BHJ: bulk heterojunction; FDTD: finite-difference time-domain; NWA: nanowire array; OPV: organic photovoltaic; P3HT: poly(3-hexylthiophene); PCE: power conversion efficiency; PML: perfect match layer.

## Competing interests

The authors declare that they have no competing interests

## Authors’ contributions

WW, XL, and LW designed the research contents and methods. WW, YZ, HD, BZ, and TS did the simulation work. XZ, NL, and YW carried out the data analysis and wrote the paper. All authors read, corrected, and approved the final manuscript.

## Authors’ information

WW got his bachelors degree in Electronic Science and Technology in 2011 at Hunan University, China. Now, he is taking his master's degree at Solid State Physics Department at Hefei Institute of Physical Science, Chinese Academy of Sciences. He is working on fabrication and characterization of semiconductor nanostructure-based applications. XL received his Ph.D. degree in Solid State Physics at Hefei Institute of Physical Science, Chinese Academy of Sciences, in Hefei in 2007. He joined the Institute of Solid State Physics (ISSP) in Hefei, China in 2007. His main research field is dedicated to the physical characterization of semiconductor nanostructures and their application in hybrid solar cells. He is an author and a coauthor of more than 30 scientific publications in journals and conference proceedings related to micro and nano systems. LW got his Ph.D. degree in Condensed Matter Physics in Solid State Physics in 2013 at Hefei Institute of Physical Science, Chinese Academy of Sciences. At present, he has a post-doctoral position at the Key Laboratory of Nanodevices and Applications, Suzhou Institute of Nano-Tech and Nano-Bionics, Chinese Academy of Sciences. He is involved in semiconductor device design and characterization of nanowires and nanoparticles of both polymeric and inorganic materials for photovoltaic applications. YZ obtained his bachelors degree in Applied Physics from China University of Petroleum in 2011. Now, he studies Solid State Physics at Hefei Institute of Physical Science, Chinese Academy of Sciences for his master's degree. What he majors in are synthesis and characterization of III-V compound semiconductor nanowires and photovoltaic applications. HD received her bachelors degree in Applied Physics in 2012 at Changchun University of Science and Technology, China. At present, she is working on fabrication and characterization of semiconductor nanostructure-based applications at Solid State Physics at Hefei Institute of Physical Science, Chinese Academy of Sciences for a master's degree. BZ obtained his master's degree in The Xinjiang Technical Institute of Physics and Chemistry, Chinese Academy of Sciences, in 2013. At present, he studies at the Solid State Physics Department at Hefei Institute of Physical Science, Chinese Academy of Sciences for a Ph.D. degree. He majors in the synthesis and characterization of semiconductor materials and semiconductor devices. TS received his Ph.D. degree at the Department of Physics of the University of Science and Technology of China in 2007. And now, he is a research associate at the Institute of Solid State Physics, Chinese Academy of Sciences. He has a background in X-ray absorption spectrum, polymer solar cells, and thin films coatings. XZ obtained his bachelors degree in Materials Science and Engineering in 2009 at Nanjing University, China. Now, he stays at Solid State Physics Department at Hefei Institute of Physical Science, China Academy of Sciences for a Ph.D. degree. He is working on fabrication and characterization of polymer semiconductor nanostructure. NL received his bachelors degree in Applied Physics in 2011 at Anhui University, China. At present, he is working on fabrication and characterization of polymer semiconductor at Solid State Physics Department at Hefei Institute of Physical Science, Chinese Academy of Sciences for his master's degree. YW obtained his Ph.D. degree from Columbia University in 1993. Now, he is a professor in Solid State Physics at Hefei Institute of Physical Science, Chinese Academy of Sciences. His research interests are wide-gap semiconductor materials, novel semiconductor devices, and semiconductor quantum structures.
